# Predicting the Potential Distribution of *Amyelois transitella* (Walker) in China Under Climate Change Using a Biomod2-Based Ensemble Model

**DOI:** 10.3390/insects17040364

**Published:** 2026-03-27

**Authors:** Shang-Lin Li, Lin Huang, Tao Yang, Yan Zhao, Bi Ding, You-Ming Hou

**Affiliations:** 1 State Key Laboratory of Agricultural and Forestry Biosecurity, Fujian Agriculture and Forestry University, Fuzhou 350002, China; lishanglin0511@163.com (S.-L.L.); huanglin0505@163.com (L.H.); yangtao11823@163.com (T.Y.); 2 Fujian Academy of Forestry, Fuzhou 350012, China

**Keywords:** *Amyelois transitella*, biological invasion, Biomod2, habitat suitability, SDMs, climate change adaptation

## Abstract

The Navel Orangeworm (*Amyelois transitella* Walker, 1863), a primary pest of nut crops native to North America, poses a significant potential threat to China’s agricultural biosecurity, yet its potential distribution dynamics under climate change remain unquantified. This study utilized the Biomod2 ensemble model platform to predict habitat suitability under current and future climate scenarios (SSP1-2.6 and SSP5-8.5). We evaluated the prediction accuracy of the ensemble model using calibration data, with TSS = 0.898 and AUC = 0.978, while spatially stratified cross-validation confirmed moderate spatial transferability to novel environments (median validation AUC = 0.60–0.75). The model identified thermal factors—Temperature Seasonality (Bio4) and the Mean Temperature of the Wettest Quarter (Bio8)—as the dominant drivers of distribution. While currently climatically suitable habitats are primarily confined to the tropical and subtropical regions of southern China, projections indicate a complex spatial shift driven by future warming: optimal southern habitats will undergo a net contraction due to heat stress, whereas low and moderately suitable areas will expand northward into key temperate agricultural areas. These results highlight that climate change will substantially alter the spatial topology of the pest’s climatic envelope, providing a critical scientific baseline of climatic suitability. These projections do not equate to realized invasion risk, which is further constrained by host availability, land use, irrigation, and human transport, offering a conservative framework for prioritizing early surveillance and optimizing quarantine measures.

## Simple Summary

*Amyelois transitella* is a destructive insect pest native to North America that damages various nut and fruit crops. Although not yet established in China, the rising international trade of agricultural products increases the risk of its introduction. This study aimed to evaluate the potential climatic suitability for this pest in China under current and future climate conditions. We used computer models to analyze environmental factors, such as temperature and rainfall, to map its potential distribution. The results indicate that temperature is the primary environmental constraint on its survival. Currently, climatically suitable habitats are largely confined to warm southern regions. However, as the climate warms, the total highly suitable area in the south is projected to contract due to heat stress, while moderately suitable areas are expected to expand northward toward temperate agricultural regions. We emphasize that these maps represent potential climatic suitability rather than realized invasion risk, as actual establishment also depends on host plants and human activities.

## 1. Introduction

Biological invasion is widely recognized as one of the major global ecological challenges of the 21st century, often leading to irreversible biodiversity loss and economic burden [1]. With the rapid expansion of international trade and global logistics networks, the speed and scale of the transboundary spread of alien species have increased significantly, posing serious threats to the ecological security and agricultural economies of various nations [2]. The Navel Orangeworm (*Amyelois transitella* Walker, 1863) (Lepidoptera: Pyralidae), a significant quarantine pest native to the Americas, has garnered global attention due to its highly destructive nature and potential expansion trends [3]. Currently widely distributed in the United States and Mexico, this pest is a primary threat to the almond, pistachio, and walnut industries in California, causing annual economic losses amounting to hundreds of millions of dollars through management costs and crop rejection [4].

*A. transitella* is particularly dangerous due to a twofold damage mechanism: it causes direct yield loss through larval feeding on fruit kernels and maintains a mutualistic relationship with the fungus *Aspergillus flavus*. This association significantly increases the risk of aflatoxin contamination in agricultural products, a potent carcinogen that renders crops unsafe for human consumption and creates severe barriers to international trade [5,6]. Notably, this species possesses strong environmental adaptability and polyphagia, feeding on a wide variety of hosts. Its larvae are highly cryptic, often burrowing deep into the fruit or nut, making them difficult to detect during visual inspections and prone to long-distance dispersal via fruits, nuts, and packaging materials [7]. While research on *A. transitella* has largely focused on its biological characteristics, pheromone-based mating disruption, and field control in its native range [8], there is a lack of systematic quantitative analysis regarding its potential habitat distribution and climatic suitability in non-native regions, particularly in China, in the context of global climate change [9,10].

Although there are currently no confirmed records of *A. transitella* colonization in China, the invasion situation is critical and warrants preemptive action [3]. On one hand, the continuous growth in the trade volume of fruit and nut products between China and the Americas significantly increases the probability of pest introduction via trade logistics [11]. On the other hand, from a climate suitability perspective, the warm and humid climate of southern China and the vast Yangtze River Basin is highly compatible with the survival environment of the pest’s native range in North America [12,13]. More importantly, widely cultivated economic crops in China, such as citrus, walnuts, jujubes, and grapes, are suitable hosts for *A. transitella*, providing ample food resources and ideal ecological niches for colonization and rapid outbreak upon introduction [14,15]. Given the severe harm it causes globally, China has proactively listed it in the Directory of Entry Plant Quarantine Pests [16]. Therefore, under the backdrop of accelerating climate change, accurately predicting its potential suitable areas and climatic changes in China is of great scientific significance for formulating proactive early warning systems and strategic control policies [17,18].

Accurate prediction of suitable habitats is a necessary component of pest risk assessment [4]. Species distribution models (SDMs) are widely used to estimate the ecological niches of alien species [19]. To limit model overfitting and spatial bias, current SDM practices emphasize the definition of accessible historical ranges (the ‘M’ area in the Biotic–Abiotic–Movement framework) [20], spatial cross-validation, and environmental collinearity screening. In this study, we utilized the Biomod2 ensemble modeling platform to evaluate the potential distribution of *A. transitella*. Specifically, we sought to calibrate a fundamental climatic niche model based on the species’ accessible range in North America [21], identify the primary climatic variables associated with its distribution, and project habitat suitability in China under current conditions and two CMIP6 future climate scenarios (SSP1-2.6 and SSP5-8.5) for the 2030s and 2050s. Because our models rely exclusively on macro-climatic and topographic predictors, we model climatic and environmental suitability. We do not claim to forecast the precise future establishment or realized invasion risk of this pest, as actual establishment is further constrained by factors such as host plant distribution, land use, irrigation practices, and human-mediated transport. Instead, these spatial projections provide a foundational climatic envelope to serve as reference data for evaluating monitoring and quarantine priorities.

## 2. Materials and Methods

### 2.1. Species Occurrence Data

**Figure 1:**
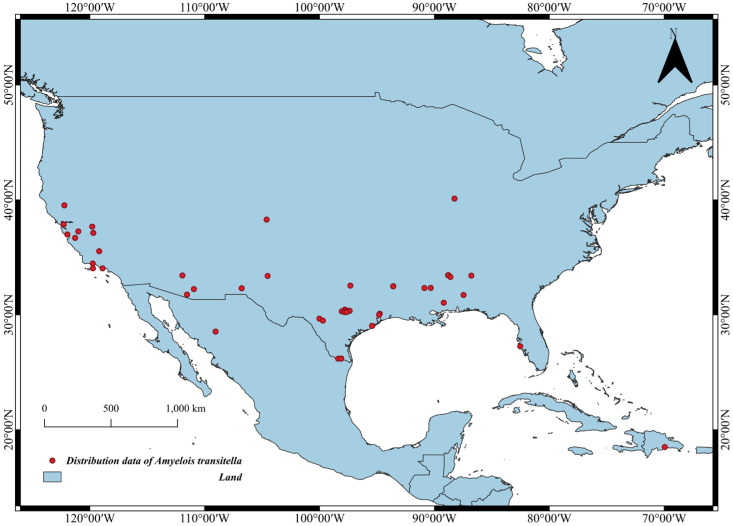
Recorded distribution points of the Navel Orangeworm (*Amyelois transitella* (Walker, 1863)) in North America.

To ensure the accuracy and reliability of species distribution predictions, occurrence records for the Navel Orangeworm (*A. transitella*) were obtained from the Global Biodiversity Information Facility (https://www.gbif.org/ (accessed on 11 February 2026)) [22]. These records cover spatial distribution data from the species’ first discovery in 1863 to the present. To temporally align the occurrence data with the baseline environmental variables, records collected prior to 1970 were excluded. Considering that *A. transitella* has not yet undergone extensive global invasion and diffusion, we compiled 170 original distribution points primarily from the United States and Central America. The quality of occurrence data is paramount for SDMs; therefore, rigorous data cleaning was performed. Sampling bias is a common issue in open-access databases, where data points are often clustered in easily accessible areas (e.g., near research stations or cities), potentially biasing the model towards the environmental conditions of those specific locations rather than the species’ true niche [23,24]. To mitigate spatial autocorrelation and sampling bias among distribution points, we enabled the filter.raster = TRUE option in the BIOMOD_FormatingData function, using climate layers as a spatial thinning mask. This approach allowed us to minimize spatial clustering by retaining only one occurrence record per grid cell of the environmental variables, ensuring a more balanced distribution of presence records across environmental gradients [25]. After this strict spatial filtering process, a total of 47 high-quality presence points were used for subsequent modeling; their geographic distribution in North America is shown in Figure 1.

### 2.2. Accessible Area and Pseudo-Absence Generation

Model calibration was restricted to the species’ accessible historical range (‘M’ region) to prevent algorithms from sampling background environments that are biologically inaccessible to the species [20]. The M polygon was defined as the North American continent between latitudes 15° N and 50° N. Background data and pseudo-absences (PAs) were constrained within this boundary. PAs were generated using the Surface Range Envelope (SRE) strategy in Biomod2 [26], which extracts points outside the 10% environmental envelope of the presence records. Five independent sets of PAs were generated (1000 points per set) to evaluate variability in background sampling.

### 2.3. Climate and Environmental Variables

This study involved two types of environmental variables: bioclimatic variables and topographic variables. Bioclimatic variables are core environmental parameters derived from monthly temperature and rainfall values to generate more biologically meaningful variables (e.g., seasonality, extreme quarters). These describe long-term climatic conditions in a specific region and are directly related to species survival thresholds, growth rates, and reproductive cycles. They primarily cover temperature and precipitation dimensions and are widely regarded as major determinants of species distribution at macro-scales. We downloaded recent (1970–2000) climate data from the WorldClim database (https://www.worldclim.org/) (Appendix B, Table A1). Considering the subsequent modeling platform and the spatial scale of the known species distribution points, we selected bioclimatic data with a spatial resolution of 5 arc-minutes (approx. 10 km at the equator), containing 19 standard variables. For topographic data, we selected elevation data with the same precision as the bioclimatic variables, as altitude serves as a proxy for localized climatic variations not captured by standard macro-climate layers.

**Figure A1:**
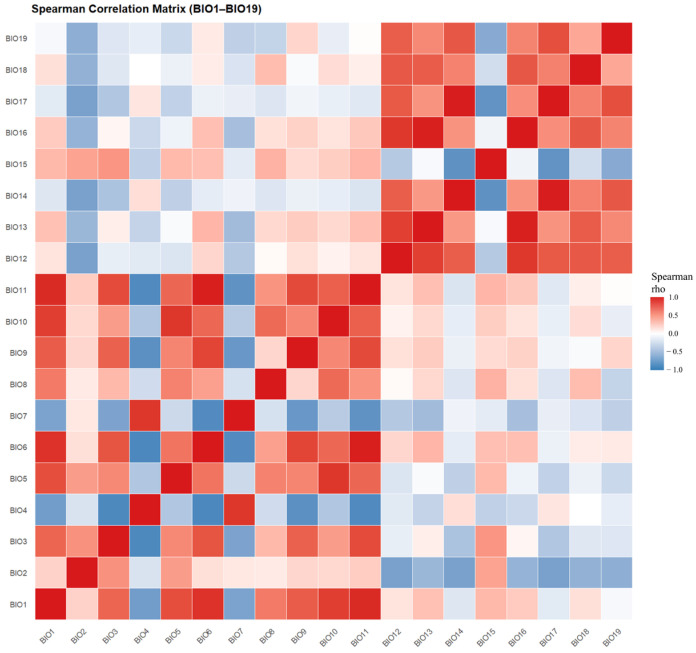
Spearman correlation matrix between 19 bioclimatic variables (bio1–bio19) used to model the potential distribution of *Amyelois transitella* (Walker, 1863).

To avoid model overfitting and instability caused by multicollinearity among predictors, 20,000 background points were randomly sampled across the defined M region [27]. A two-step reduction strategy was implemented using the usdm package v2.1-7 [28]. First, variables were clustered based on a Spearman rank correlation threshold of |r| ≥ 0.7. Second, a Variance Inflation Factor (VIF) step-wise selection was applied to the representative variables, excluding those with a VIF ≥ 5 (Appendix A, Figure A1 and Appendix B, Table A2) It is important to note that this variable screening procedure was conducted on the entire background dataset within the M region prior to assigning the spatial cross-validation folds, rather than being fully nested within each fold. Additionally, the elevation variable was introduced as a topographic factor, extracted from the global Digital Elevation Model (DEM) provided by WorldClim (https://www.worldclim.org/) [29]. We unified all environmental variables to the same spatial resolution and projection extent to ensure data consistency and avoid prediction bias caused by data source heterogeneity.

Future climate data were selected from the CMIP6 simulation results, using the BCC-CSM2-MR climate system model developed by the Chinese Academy of Meteorological Sciences [30]. Compared to the previous CMIP5 generation, CMIP6 models incorporate more complex physical processes and higher resolution, offering improved simulation of East Asian climate patterns [31]. This model has demonstrated good simulation stability and regional adaptability in multiple climate assessments [32]. Two typical Shared Socioeconomic Pathways (SSPs) scenarios were selected—SSP1-2.6 (low-emission/sustainability pathway) and SSP5-8.5 (high-emission/fossil-fueled development)—representing different global greenhouse gas emission scenarios and potential warming intensities [33]. This study focused on analyzing two time periods: 2021–2040 (near future) and 2041–2060 (mid-future) to assess the dynamic impact of future climate change on potential species distribution patterns [34]. We hope the results will be used for proactive risk assessment and prediction of key climate variables for this species.

### 2.4. Biomod2 Modeling Procedure

This study established a suitable habitat prediction model for *A. transitella* based on Biomod2 v4.3-4-5 [35,36], a leading ensemble modeling approach in R. To reduce the uncertainty and overfitting risks associated with relying on any single algorithm, an ensemble modeling approach was adopted. This method integrates multiple classical niche models, including regression methods (GAM, MARS), machine learning methods (ANN, RF, GBM, XGBOOST), and classification methods (CTA, FDA, SRE, MAXNET) [37]. This diversity enhances prediction stability and generalization capability by averaging out the specific biases of individual techniques (an overview of modeling algorithms used in Biomod2 and their corresponding R packages v4.5.2 is provided in Appendix B, Table A3). Furthermore, using multi-algorithm ensembles can effectively identify sources of model prediction uncertainty and reduce the impact of model structural bias [38]. Relevant studies indicate that Biomod2-based ensemble methods possess high accuracy and ecological rationality in predicting species-suitable habitats under climate change contexts.

Model calibration employed the cross-validation method to ensure that the results were not dependent on a specific subset of data. To evaluate model transferability, a spatially stratified cross-validation approach was used. Data were partitioned into four latitudinally stratified folds (strat = ‘y’), testing the models on spatially distinct subsets [39]. Algorithm hyperparameters were set using the “bigboss” tuning strategy provided within the package. This hyperparameter tuning is nested in each space fold (calib.lines = cv.s). Model performance was evaluated using the True Skill Statistic (TSS), Area Under the Receiver Operating Characteristic Curve (ROC/AUC), and Cohen’s Kappa index. These indices are widely used in niche modeling to measure model stability and predictive power [40,41]. To provide a transparent and rigorous assessment, we explicitly distinguished between calibration metrics (which indicate model fit to the training data) and spatially stratified validation metrics (which serve as the primary indicator of the model’s spatial transferability to novel environments). TSS is particularly useful as it accounts for both omission and commission errors and is independent of prevalence. During model training, var.import = 3 was set, and variable importance for each single model was calculated through two random permutations to quantify the contribution of environmental factors to model output [42].

After single-model training was completed, the BIOMOD_EnsembleModeling function was used for model integration. Ensemble modeling approach included six methods: EMmedian (median of probabilities over the selected models), EMmean (mean of probabilities over the selected models), EMwmean (weighted mean of probabilities over the selected models), EMca (committee averaging over the selected models), EMci (confidence interval around the mean of probabilities of the selected models) and EMcv (coefficient of variation (sd/mean) of probabilities over the selected models). To ensure model quality, predictions from single models were only included in the weighted calculation if their spatially stratified validation TSS ≥ 0.60 [43]. The weighting process used the decay = ‘proportional’ parameter, allowing model weights to decrease proportionally according to performance metrics to reduce the influence of lower-accuracy models. In the ensemble stage, the importance of variables to the overall prediction result was evaluated through three random permutations to quantify the contribution of environmental factors and reduce model uncertainty.

The final ensemble modeling approach was projected onto current climate conditions and four future climate scenarios. In the final ensemble model, projected habitat suitability is automatically re-scaled by Biomod2 to an integer range of 0 to 1000 to optimize computational efficiency and data storage. A clamping mask (build.clamping.mask = TRUE) was enabled during model projection to quantify uncertainty in extrapolation areas where future or projected environmental conditions might exceed the range of training data. In the resulting clamping mask rasters, pixel values ranging from 0 to 4 indicate the number of environmental variables that fall outside their respective calibration ranges. This effectively represents the extrapolation risk, which we classified from “None” (0 variables out of range) to “Very High” (four variables out of range). The bm_PlotRangeSize function was used to calculate changes in future suitable habitats relative to the current situation and generate distribution visualization maps for the globe.

### 2.5. Eco-Zone Classification and Threshold Definition

We imported the ensemble-optimized suitable habitat projections into QGIS 3.42.2 for post-processing and analysis. In the Biomod2 ensemble habitat suitability raster projection, suitability levels ranged from integers 0 to 1000. To establish a rigorous ecological threshold for determining binary habitat suitability, we applied the threshold that maximizes the True Skill Statistic (maxTSS). According to the results of the Biomod2 analysis, we used the “get_evaluations” command to check, and the threshold obtained was 509. This indicates that, based on this binary classification method, areas with values greater than 509 are suitable habitats, while those with values less than 509 are unsuitable habitats. Meanwhile, based on the Jenks natural breaks method, habitat suitability was classified into four categories—unsuitable, low suitability, moderate suitability, and high suitability [44], which were used for visualizing the suitable habitat for *A*. *transitella*. Our classification thresholds were set as follows: unsuitable areas [0–271), low-suitability areas [271–562), moderate-suitability areas [562–743), and high-suitability areas [743–1000]. Area calculations were performed in QGIS using the Asia North Albers Equal Area Conic projection.

## 3. Results

### 3.1. Model Accuracy and Spatial Transferability

**Figure 2:**
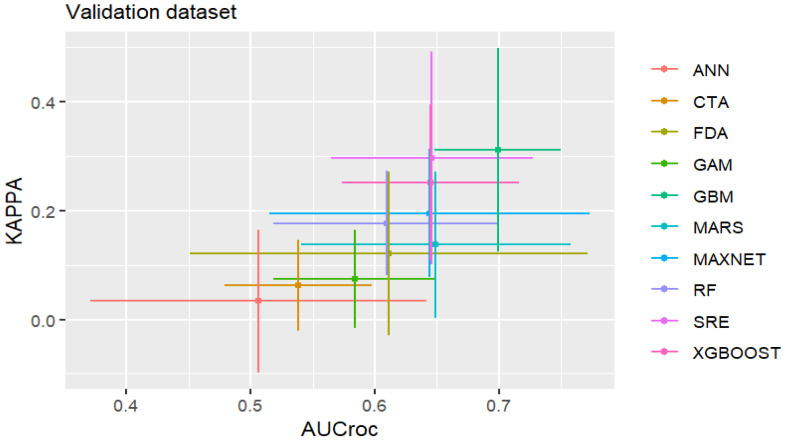
Mean evaluation metrics (AUC, Kappa) for all algorithms on the validation dataset.

**Figure A2:**
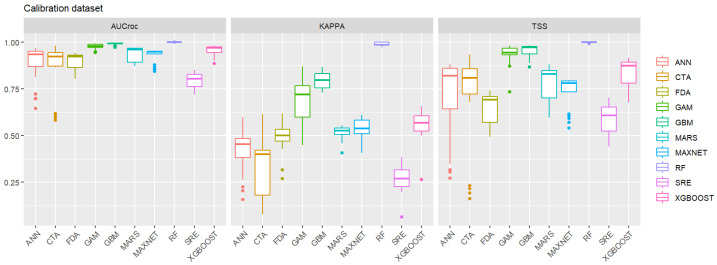
Distribution of evaluation metrics by algorithm on the calibration dataset.

**Figure A3:**
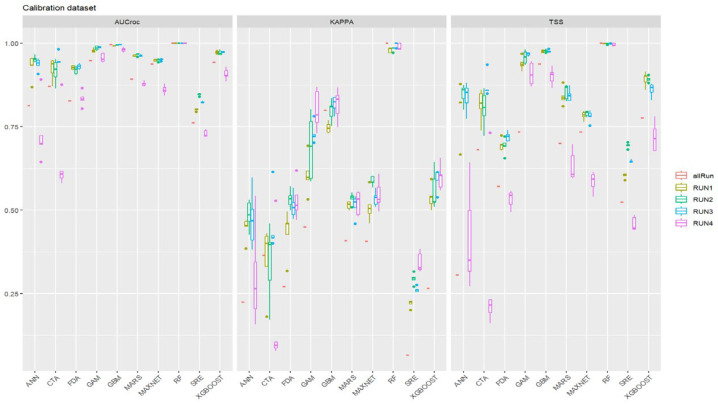
Variability of evaluation metrics across runs for each algorithm.

The evaluation metrics indicated variable performance across the tested algorithms when subjected to spatially stratified cross-validation [45]. During the calibration phase, which evaluated model fit to the training data, complex machine learning algorithms such as Random Forest (RF), XGBOOST, and Generalized Additive Models (GAM) produced exceptional discrimination metrics (median calibration AUC > 0.95, Kappa > 0.75) (Appendix A, Figure A2 and Figure A3). However, testing on the spatially segregated validation folds provided a more conservative and realistic measure of model transferability to novel geographic spaces [39]. Under these strict spatial constraints, the spatially stratified validation metrics revealed that algorithms including RF, GBM, and GAM demonstrated robust generalization capabilities, maintaining median validation AUC scores between 0.60 and 0.75 (Figure 2). To mitigate the algorithmic biases and variance observed in individual models, the final ensemble model was constructed only of models meeting the spatial validation TSS > 0.60 threshold. This ensemble approach significantly stabilized predictive performance. When evaluating the final ensemble model against the aggregated calibration data, it yielded an excellent model fit with an AUC of 0.978 and a TSS of 0.898 based on the calibration dataset. We rely on the spatially stratified validation metrics as the primary indicator of the model’s performance when projecting into new geographical areas. High calibration metrics only indicate that the model’s fit is ideal, not that the prediction accuracy is high. This ensemble approach stabilized predictive performance, yielding a consensus projection suitable for spatial analysis.

### 3.2. Environmental Variable Importance and Response Curves

**Figure 3:**
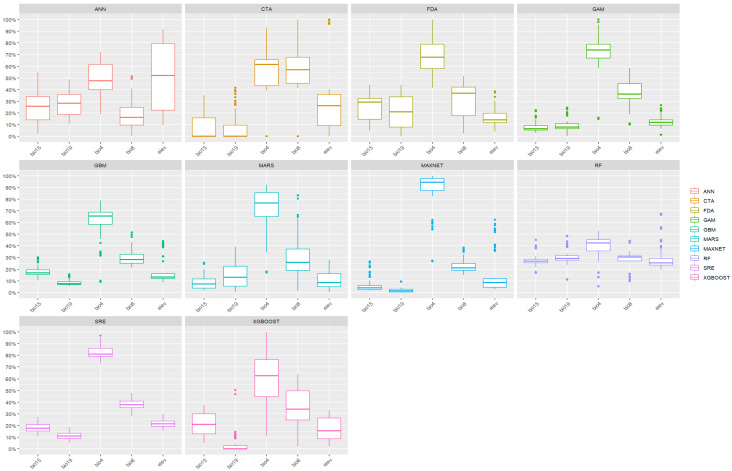
Variable importance of selected environmental predictors across algorithms..

**Figure A4:**
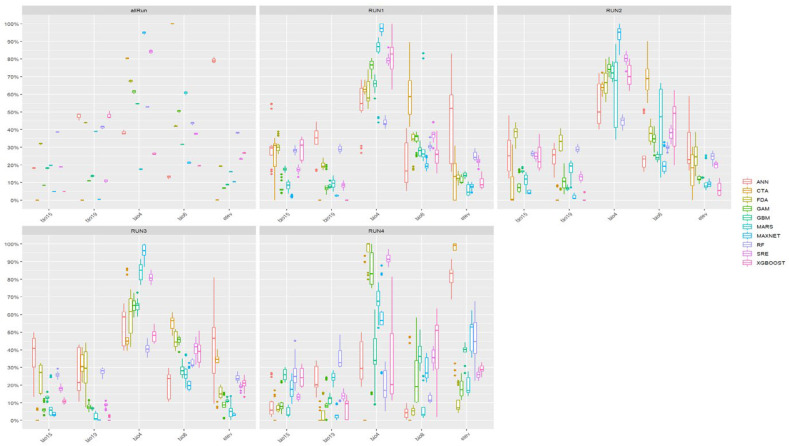
Variation in variable importance across algorithms and cross-validation runs.

**Figure A5:**
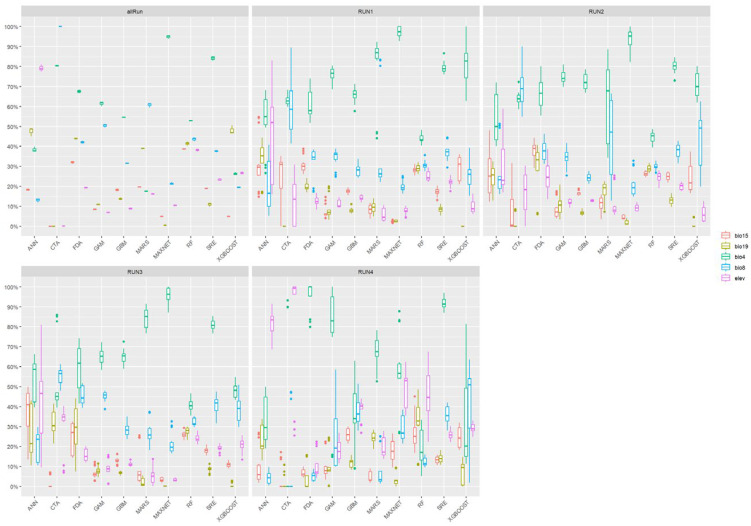
Algorithm-specific differences in environmental variable contributions.

Based on the permuted variable importance metrics extracted from the final ensemble model, thermal parameters emerged as the dominant constraints on the potential distribution of *A. transitella*. Temperature Seasonality (Bio4) and Mean Temperature of the Wettest Quarter (Bio8) exhibited the highest relative contribution scores across the majority of the constituent algorithms, collectively accounting for the largest proportion of model variance (Figure 3). Elevation (elev), Precipitation Seasonality (Bio15), and Precipitation of the Coldest Quarter (Bio19) contributed moderately to the predictive outputs. However, as variable screening was not fully nested within the spatial cross-validation, these rankings should be interpreted cautiously as robust climatic. (Appendix A, Figure A4 and Figure A5).

The univariate response curves, which isolate the modeled relationship between individual environmental predictors and habitat suitability while holding other variables at their mean values, provided specific physiological thresholds (Figure 3). The probability of species presence exhibited a strict negative relationship with Temperature Seasonality (Bio4). Suitability probabilities reached their maximum where annual temperature variance was minimized and declined sharply as temperature seasonality increased, suggesting that environments characterized by extreme seasonal temperature fluctuations are unfavorable for establishment. Conversely, the response to the Mean Temperature of the Wettest Quarter (Bio8) demonstrated a unimodal distribution. Suitability peaked distinctly within a thermal window of approximately 20 to 25 °C. The response curve for Elevation (elev) indicated an inverse relationship with suitability, which remained highest near sea level and decreased steadily as elevation increased, establishing the species primarily as a lowland inhabitant. Regarding moisture requirements, Precipitation Seasonality (Bio15) displayed a positive association with habitat suitability. The model indicated higher presence probabilities in regions experiencing pronounced precipitation variance, such as distinct wet and dry seasons. Similarly, Precipitation of the Coldest Quarter (Bio19) contributed to the characterization of necessary overwintering conditions, showing threshold responses to winter moisture availability.

**Figure A6:**
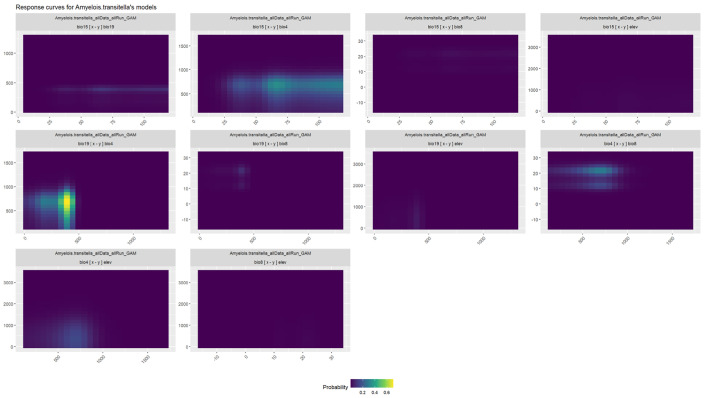
Bivariate response surfaces showing interaction effects among key environmental predictors.

Furthermore, the bivariate response surfaces revealed substantial interaction effects between thermal stability and moisture variability (Appendix A, Figure A6). The highest suitability probabilities consistently emerged under specific combinations: low Temperature Seasonality (Bio4) coupled with high Precipitation Seasonality (Bio15). This quantitative interaction aligns with the climatic profile of Mediterranean-type ecosystems, characterized by mild, wet winters and warm, dry summers, underscoring the integrated nature of the climatic drivers governing the species’ distribution.

### 3.3. Global Potential Geographic Distribution Under Current Climate

**Figure 4:**
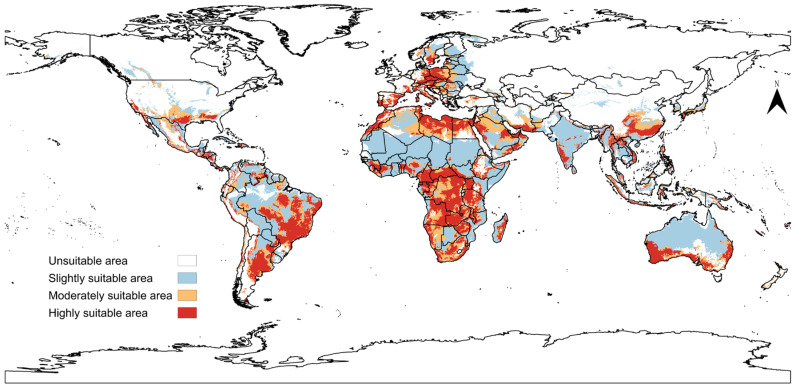
Predicted global potential distribution of *Amyelois transitella* (Walker, 1863) based on SDMs.

**Figure A7:**
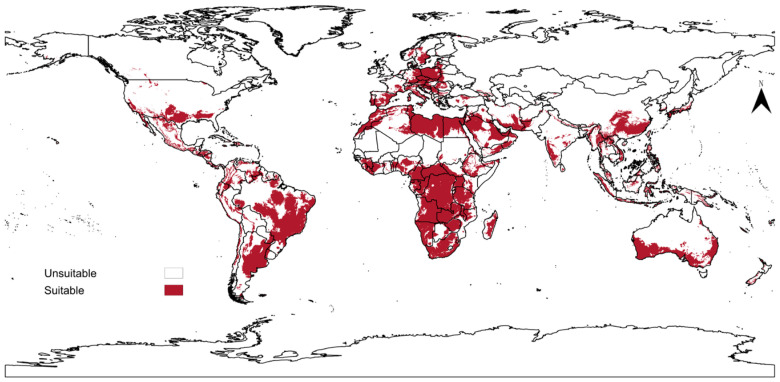
Predicted global potential distribution of *Amyelois transitella* (Walker, 1863) based on the Biomod2 ensemble model. The binary habitat suitability (unsuitable and suitable) was determined based on the maximum True Skill Statistic (maxTSS) threshold.

Under current climate conditions, based on the maxTSS threshold, the total globally suitable area for *A. transitella* was estimated to be approximately 34,084,586.06 km^2^ when calculated using the Equal Earth projection with the EPSG:8857 coordinate reference system (Appendix A, Figure A7). The global spatial projection successfully reproduced the known occurrence patterns of *A. transitella* within its native and historical ranges in North America (Figure 4). Visualizing the suitability gradients reveals that the model assigned the highest suitability probabilities to the western United States, precisely capturing the core agricultural regions of California, such as the Central Valley, which are characterized by distinct wet and dry seasons. Similarly, suitable habitats extended into the southwestern United States and specific northern regions of Mexico, corroborating the species documented physiological tolerance limits.

**Figure A8:**
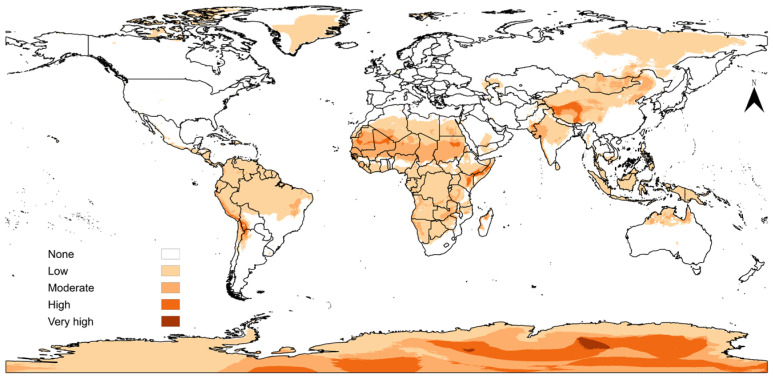
Extrapolation risk of the *Biomod2* ensemble model for *Amyelois transitella* (Walker, 1863) globally.

Besides the North American continent, the binary model also identified several discontinuous geographical regions globally with similar environmental conditions, indicating substantial climatic suitability for potential colonization that if *A. transitella* introduced. In South America, high-suitability areas were concentrated along the central coast of Chile. Extensive areas of high climatic suitability were identified across the Mediterranean Basin, encompassing the southern coastal regions of Spain, Italy, and Greece, as well as northern African countries bordering the Mediterranean Sea, such as Morocco and Tunisia. Furthermore, the model projected isolated suitable areas in the Western Cape province of South Africa and the southwestern coastal regions of Australia. The evaluation of extrapolation uncertainty using the clamping mask (Appendix A, Figure A8) demonstrated that these globally discontinuous suitable regions generally exhibit low extrapolation risk. If we need to make climate suitability predictions for “Very high” areas, we must make conservative inferences. This is because reasoning beyond the training environment variables was forced in the projection results of these areas. The global suitable regions for *A. transitella* identified by maxTSS are all in the “low” areas.

### 3.4. Potential Distribution in China Under Current Climate

**Figure 5:**
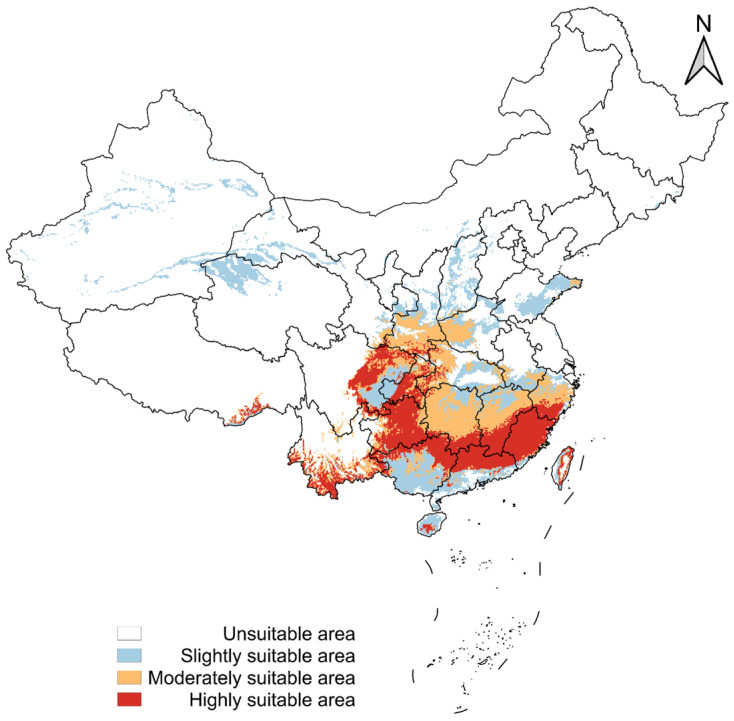
Predicted potential distribution of *Amyelois transitella* (Walker, 1863) in China based on SDMs.

**Figure A9:**
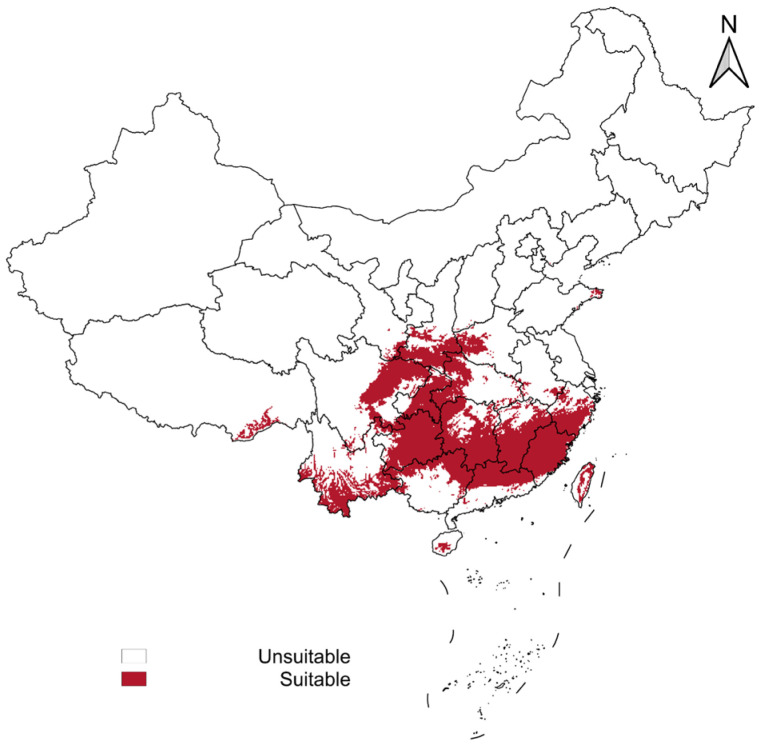
Predicted potential distribution of *Amyelois transitella* (Walker, 1863) in China based on the Biomod2 ensemble model. The binary habitat suitability (unsuitable and suitable) was determined based on the maximum True Skill Statistic (maxTSS) threshold.

Based on the maxTSS threshold, the current modeled suitable habitat for *A. transitella* in China encompasses an estimated total area of approximately 1,347,998.24 km^2^ when calculated using the Asia North Albers Equal Area Conic projection (Appendix A, Figure A9). This indicates a concentrated distribution pattern restricted primarily to the southern and southeastern regions (Figure 5). Visualizing the suitability gradients based on the Jenks classification (Figure 5) reveals that the optimal climatic zones are predominantly clustered in the tropical and subtropical provinces. These include continuous patches across Guangdong, Guangxi, Hainan, southern Fujian, and the southern extremities of Yunnan province. These regions present a current climatic profile characterized by mild winters and moderate thermal stability, aligning with the species’ requirement for favorable wet-quarter temperatures (Bio8).

Radiating outward from these core optimal zones, the suitability gradient gradually decreases to form a geographical buffer that encompasses major portions of Jiangxi, Hunan, Guizhou, and central Fujian. These intermediate regions represent areas where environmental conditions permit survival and development but may occasionally subject populations to suboptimal temperature fluctuations. The northern boundary of marginal climatic suitability extends toward the middle and lower reaches of the Yangtze River Basin, incorporating parts of Zhejiang, Anhui, and Hubei provinces.

**Figure A10:**
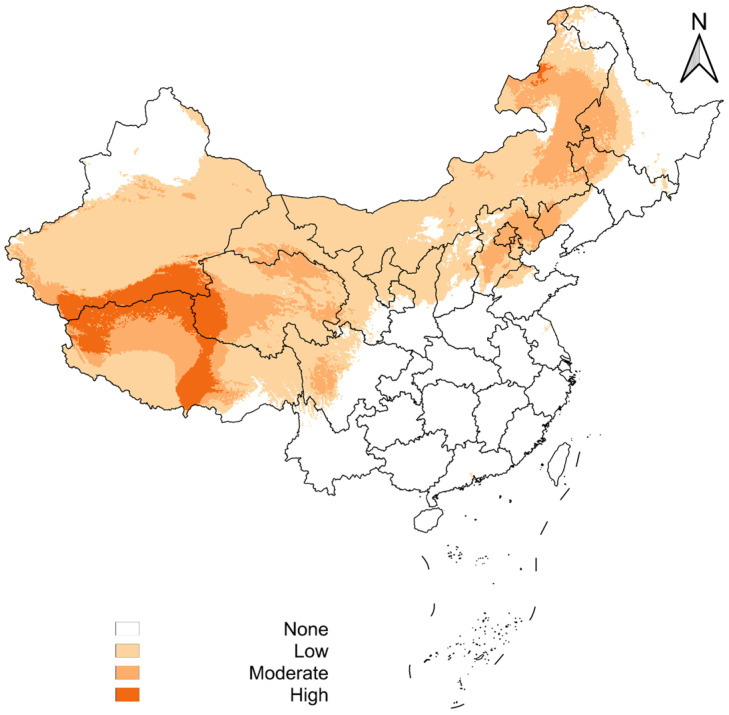
Extrapolation risk of the Biomod2 ensemble model for *Amyelois transitella* (Walker, 1863) in China.

The binary model classifies the vast majority of the Chinese landmass as currently unsuitable for *A. transitella* (Appendix A, Figure A9). This extensive unsuitable area encompasses the entire northern, northwestern, and northeastern geographic regions, including Xinjiang, Gansu, Inner Mongolia, and the Northeast China Plain, as well as the high-altitude Tibetan Plateau. The exclusion of the species from these northern and western territories is primarily driven by elevated Temperature Seasonality (Bio4) and severe winter cold stresses (Bio19), which exceed the physiological overwintering capacities and seasonal development thresholds established for this pest. Furthermore, an assessment of extrapolation uncertainty using the clamping mask reveals that the currently suitable regions in southern China exhibit very low extrapolation risk (Appendix A, Figure A10). This confirms that the environmental conditions in these susceptible southern provinces fall well within the calibration data range of the model, underscoring the reliability of the climatic suitability projections in these key agricultural areas.

### 3.5. Potential Distribution in China Under Future Climate Conditions

**Figure A11:**
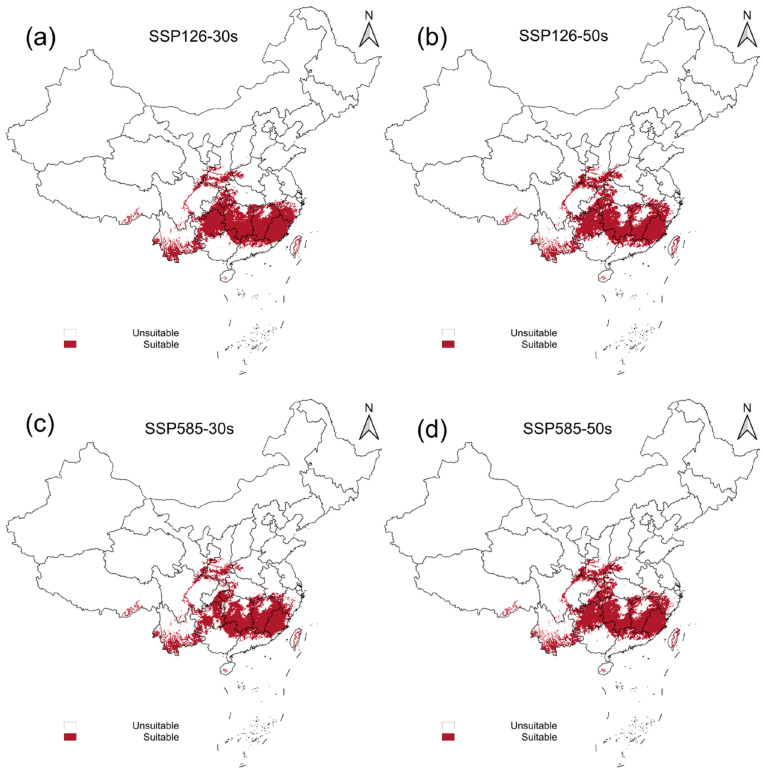
Predicted potential distribution of *Amyelois transitella* (Walker, 1863) in China under future climate scenarios using the Biomod2 ensemble model. (**a**) SSP1-2.6 in the 2030s; (**b**) SSP1-2.6 in the 2050s; (**c**) SSP5-8.5 in the 2030s; (**d**) SSP5-8.5 in the 2050s. The binary habitat suitability (unsuitable and suitable) was determined based on the maximum True Skill Statistic (maxTSS) threshold.

Projections extending into future climate scenarios reveal complex spatial range dynamics, characterized by a non-uniform geographic shift rather than a simple radial expansion. Based on the maxTSS threshold, the total suitable area in China is projected to experience a continuous net contraction due to future warming (Appendix A, Figure A11).

**Figure 6:**
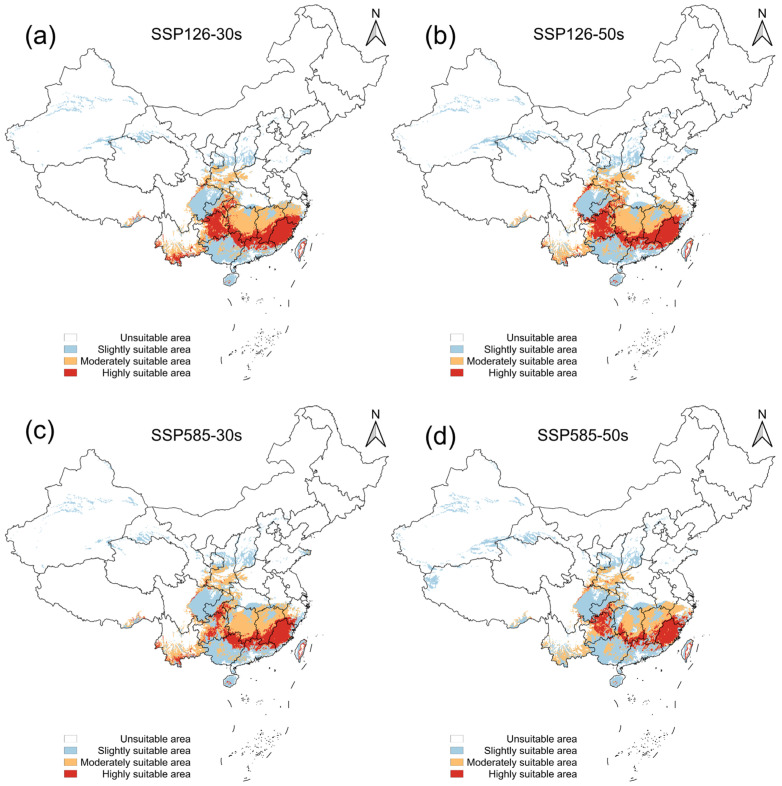
Predicted potential distribution of *Amyelois transitella* (Walker, 1863) in China under future climate scenarios predicted by Shared Socioeconomic Pathways (SSPs). (**a**) SSP1-2.6 in the 2030s; (**b**) SSP1-2.6 in the 2050s; (**c**) SSP5-8.5 in the 2030s; (**d**) SSP5-8.5 in the 2050s.

Under the low-emission SSP1-2.6 scenario, representing a trajectory of moderate climatic warming, the total suitable habitat is projected to decrease from the current baseline of approximately 1,347,998.24 km^2^ to 1,087,201.05 km^2^ by the 2030s, and further contract to 1,048,551.97 km^2^ by the 2050s. Visualizing the suitability gradients under this scenario (Figure 6) shows a steady decline of the highly suitable core areas in the extreme southern provinces. Concurrently, marginal and moderately suitable areas demonstrate an expansive trend, indicating an initial northern shift of the geographical boundary.

These bidirectional spatial shifts become significantly more pronounced under the high-emission SSP5-8.5 scenario. As regional warming intensifies, the total suitable area is projected to drop sharply to 968,185.64 km^2^ by the 2030s, ultimately collapsing to 819,801.94 km^2^ by the 2050s. The suitability gradient maps (Figure 6) reveal that the optimal southern habitats undergo severe degradation, while lower-suitability areas expand to propel the northern edge of the potential distribution past the traditional Qinling Mountains to Huaihe River climate divide. Because these projections are derived from a single GCM, it must be emphasized that these specific area metrics represent potential climatic trajectories and should not be viewed as precise, deterministic forecasts of future spread.

**Figure A12:**
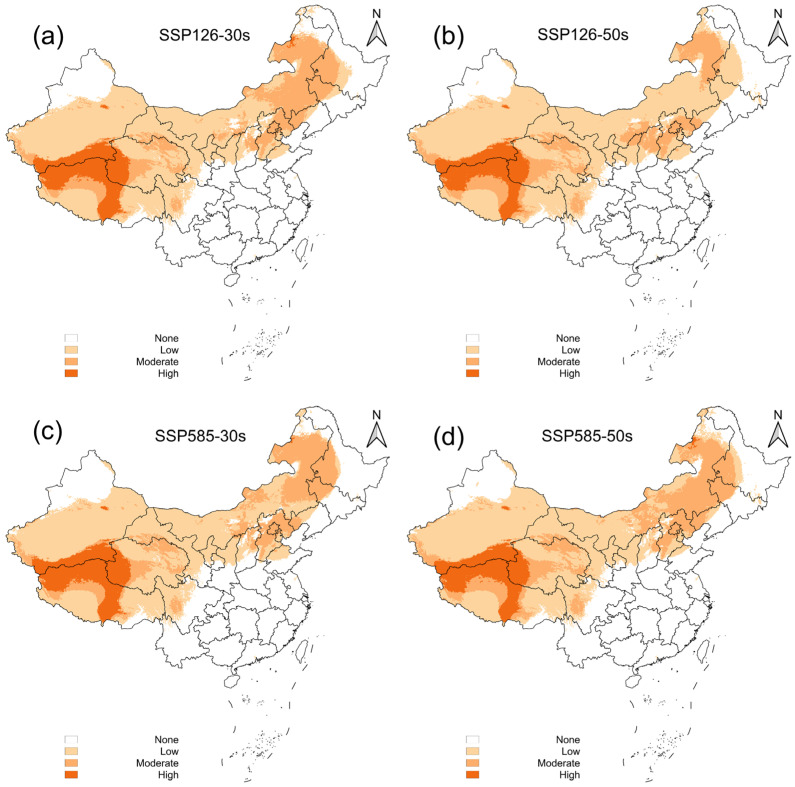
Extrapolation risk of the Biomod2 ensemble model for *Amyelois transitella* (Walker, 1863) in China under future climate scenarios. (**a**) SSP1-2.6 in the 2030s; (**b**) SSP1-2.6 in the 2050s; (**c**) SSP5-8.5 in the 2030s; (**d**) SSP5-8.5 in the 2050s.

Finally, an assessment of extrapolation uncertainty using the clamping mask provides crucial context for these future projections (Appendix A, Figure A12). As climate warming intensifies, particularly under the SSP5-8.5 scenario in the 2050s, the extrapolation risk progressively increases in certain northern and western marginal areas. This indicates that future environmental conditions in these newly suitable edge regions may locally exceed the calibration data range of the model. If the climate suitability of *A. transitella* in the future is to be inferred, the range of suitability and the results of the clamping mask analysis need to be compared to ensure that the extrapolated risk is below “Low”. Nevertheless, the core regions undergoing significant suitability contraction in southern China maintain low to moderate extrapolation risk, ensuring the reliability of the predicted habitat loss trends in these primary agricultural areas.

## 4. Discussion

### 4.1. Methodological Discussion

Species distribution models fundamentally rely on the assumption that the occurrence records used for calibration adequately represent the species’ environmental tolerances [19]. However, these models are frequently subject to uncertainties originating from spatial sampling bias and the violation of independence among presence points [27]. To provide a realistic assessment of model performance, a primary methodological advancement in our study was the implementation of a spatially stratified cross-validation approach (strat = ‘y’) rather than conventional random data splitting [45]. Traditional random partitioning often leads to inflated performance metrics because the training and testing datasets share spatial autocorrelation, essentially allowing the model to memorize local landscape features rather than learning true physiological boundaries [39]. By partitioning the data into latitudinally discrete blocks, our spatial cross-validation explicitly tested the algorithms’ capacity to extrapolate into novel geographic spaces. While this approach inevitably produced more conservative evaluation metrics during the validation phase, it provides a more realistic estimation of the model’s transferability across continents [46].

However, it is important to acknowledge a limitation regarding our variable selection process. In the present workflow, environmental variable screening was conducted prior to fold-wise model fitting, based on the M-constrained environmental dataset, rather than being repeated independently within each training fold of the spatial cross-validation. Our model hyperparameter tuning was rigorously nested within each spatial cross-validation fold (calib.lines = cv.s), which effectively prevented data leakage during the algorithm optimization phase. While this nested tuning strengthens the robustness of our models, the non-nested variable screening design may still retain some risk of optimistic variable-selection stability. Consequently, to avoid making absolute assertions regarding precise variable importance hierarchies, future work should implement fully nested fold-wise predictor screening to further strengthen inference.

Furthermore, managing sampling bias is a critical limitation in broad-scale SDMs [27]. In our study, we addressed this by restricting the background generation to the historically accessible ‘M’ area and applying rigorous spatial thinning (filter.raster = TRUE). This thinning process mitigates the over-representation of occurrences in highly sampled regions, such as agricultural valleys in California [47]. However, we acknowledge the limitation that an explicit target-group background or a continuous spatial bias surface was not integrated into the modeling pipeline. While spatial thinning effectively enforces uniform geographic sampling density, an explicit bias surface derived from the sampling effort of closely related taxa could potentially further decouple the climatic signal from human observation bias [48].

Additionally, our fundamental niche model relies exclusively on macro-climatic predictors and elevation [21]. The exclusion of non-climatic variables, such as high-resolution land-use classifications, explicit host plant distributions, and anthropogenic factors like human accessibility and irrigation density, represents a conscious methodological trade-off [49]. However, these factors can affect the establishment of the species’ population. At a global macro-scale, climate sets the absolute upper and lower boundaries for species survival. Nevertheless, in highly modified agricultural landscapes, practices such as intensive orchard irrigation can locally buffer extreme macro-climatic conditions, effectively creating micro-refugia [50]. The absence of these micro-scale predictors means our projections should be interpreted as maps of fundamental climatic suitability rather than realized micro-habitat occupancy. Future modeling iterations aiming for orchard-level precision must seek to integrate these high-resolution anthropogenic and land-use layers to capture the full complexity of agricultural ecosystems.

To further address the structural uncertainties inherent in different mathematical algorithms, our study utilized some ensemble approaches. Individual algorithms vary significantly in their complexity and their tendency to fit complex response curves. Machine learning techniques like Random Forest and XGBoost excel at capturing non-linear interactions but are prone to overfitting [51,52], whereas regression-based models like GAM provide smoother, more generalized responses [53]. By aggregating only the models that met a strict performance threshold and weighting them by their predictive accuracy, the ensemble framework effectively marginalized the extreme predictions of any single algorithm, yielding a stabilized and robust consensus projection. Additionally, we employed a clamping mask diagnostic tool to transparently identify extrapolation uncertainty, helping us appropriately interpret the reliability of projections in novel environments [54]. However, the accuracy of future distribution changes for *A. transitella* depends on the model and scenario. Our future projections are based on a single global climate model (BCC-CSM2-MR), and the analysis results have certain spatiotemporal limitations. Because integrating additional climate models was not yet integrated, our conclusions regarding future shifts remain conservative. We emphasize that the specific magnitude of geographic change is highly model-dependent and scenario-dependent, meaning our maps and precise area estimates should be viewed as potential climatic trajectories rather than precise geographical forecasts rather than deterministic geographical forecasts [55].

### 4.2. Impact of Global Climate Change on Habitat Suitability and Key Environmental Drivers

We evaluate the key environmental drivers that biologically constrain the distribution of *A. transitella*. Our rigorous collinearity screening and ensemble modeling identified Temperature Seasonality (Bio4) and the Mean Temperature of the Wettest Quarter (Bio8) as the primary macro-climatic determinants. *A. transitella* may adapt to Mediterranean-type climates and lacks an obligate physiological diapause. Instead, it overwinters as mature larvae within unharvested host nuts [56], developing continuously at a reduced rate throughout the winter provided that temperatures remain above critical developmental thresholds. Climate warming not only expands suitable areas but also alters the relative influence of environmental factors [57]. Currently, Bio4 is the primary limiting factor for *A. transitella* survival, but as winters become warmer, this limitation will weaken, potentially shifting the northern boundary of the species’ distribution deep into the temperate zone [58].

Global climate change, particularly under the high-emission SSP5-8.5 scenario, drastically alters these key environmental drivers. The increased frequency of extreme summer heat may cause heat stress to larvae and adults. While larvae are more sensitive due to limited mobility, adults may also experience shortened lifespans, reduced mating activity, and decreased oviposition rates under prolonged high temperatures [59]. In the southern provinces (e.g., Guangdong, Guangxi), the projected increase in summer temperatures combined with monsoon moisture will likely push Bio8 beyond the species’ upper physiological tolerance limit. This extreme heat stress is anticipated to disrupt larval development and promote lethal entomopathogenic infections, directly driving the severe contraction of the suitability core areas observed in our models.

### 4.3. Climatic Suitability and Surveillance Priorities of Amyelois transitella in China’s Potential Distribution Areas

Under current climatic conditions, the Biomod2 ensemble model predicts that suitable habitats for *A. transitella* in China are mainly distributed in parts of South and Southwest China. *A. transitella* is a highly polyphagous pest, causing severe economic damage to almonds, pistachios, walnuts, figs, and pomegranates in its native range. The potential distribution pattern is jointly influenced by winter cold, habitat fragmentation, and latitudinal limits, which is largely consistent with patterns of other Lepidopteran pests distributed in China [60]. Although there are no local records of *A. transitella* yet, its severe outbreaks in North America and damage it causes to nut and fruit crops highlight the importance of early warning and biosecurity preparedness [7].

The future climatic changes are particularly noteworthy for China’s agricultural zones. The predicted expansion into the Yangtze River Basin and North China Plain places the pest’s potential climatic envelope in direct proximity to major walnut, grape, and jujube production areas [8]. These specific geographic areas constitute China’s primary production of temperate fruits and nuts, representing a massive and continuous reservoir of potential host plants [61]. While our models map climatic suitability rather than realized invasion risk, since true invasion depends on human transport and local orchard dynamics, the spatial overlap between these emerging suitable climates and major crop cultivation areas warrants cautious attention. Given that walnuts are an established and preferred host for *A. transitella* in North America, the climatic change in the pest’s potential distribution directly toward China’s walnut-producing provinces poses a potential challenge to domestic agricultural security. The invasion biology of polyphagous insects often involves rapid host-shifting when introduced into novel, resource-rich ecosystems [62].

When an invasive pest encounters new flora that offer structural similarities to its native hosts, it can adapt swiftly, particularly in the absence of coevolved natural enemies. Consequently, the invasion risk in these northern and central Chinese regions cannot be understated. Although currently classified merely as low- to moderate-climatic-suitability areas based on macro-climate gradients, the dense availability of susceptible rosaceous and nut crops within these areas could theoretically support population establishment if the pest is via human transport and micro-climatic barriers are overcome. The spatial convergence of increasing climatic hospitality due to warming winters and an abundance of novel and viable hosts dictates that these regions should be treated as priority zones for early surveillance and phytosanitary inspections in future agricultural planning [63].

### 4.4. Control Strategies for Amyelois transitella in China

Port quarantine is the first line of defense against potential introduction. The listing of *A. transitella* in the “Catalogue of Quarantine Pests for Import Plants to China” marks official recognition of its invasion risk. Therefore, it is recommended to implement high-standard entry quarantine and fumigation procedures for unprocessed nuts (almonds, pistachios, walnuts), citrus fruits, and dried fruit products imported from high-risk regions such as the United States. Advanced molecular detection techniques, such as real-time PCR or DNA barcoding, should be employed to rapidly identify larvae intercepted at ports, which are often morphologically indistinguishable from native species [64,65,66]. Furthermore, byproducts including nut shells, fruit residues, and wood packaging materials should be regulated, as they may harbor cryptic pest stages. Imports of untreated nut shell waste and dried fruit mixtures should be restricted, coupled with improved quarantine protocols (fumigation and heat treatment). Major entry ports (e.g., Shanghai, Guangzhou, Tianjin) should increase inspection frequency and establish species-specific identification processes for high-risk commodities like almonds, figs, and raisins [67].

For provinces predicted to have moderate to high suitability (e.g., Jiangxi, Hunan, Fujian, and Guangdong), proactive early warning and emergency response frameworks are crucial. Local agricultural departments should formulate emergency plans combining orchard structure, climatic conditions, and invasion probability. Once detected, containment strategies involving pheromone mating disruption [14], field inspection, orchard sanitation, and targeted chemical control should be rapidly implemented [68]. Long-term strategies should include Integrated Pest Management (IPM) education for growers in high-risk areas to recognize early signs of infestation.

## 5. Conclusions

This study employed a Biomod2-based ensemble modeling approach to predict the potential distribution of *A. transitella* and assess its response to future climate change. Results indicate that current suitable habitats are mainly distributed in the Americas, with potential distribution areas also in the Mediterranean, South Asia, southern Africa, and parts of Oceania, demonstrating the species’ strong climatic adaptability. In China, suitable habitats are currently confined to tropical and subtropical regions in the south, limited by high Temperature Seasonality and low winter temperatures in the north. Temperature-related factors, specifically Temperature Seasonality (Bio4) and the Mean Temperature of the Wettest Quarter (Bio8), were identified as key environmental drivers. Although in current climate, high-suitability areas in China are concentrated in southern provinces, growing agricultural trade with the Americas increases the risk of accidental introduction. Model predictions suggest that under future warming scenarios, particularly under the high-emission scenario (SSP5-8.5), high-suitability areas in southern China will decrease due to heat stress, while low- and moderate-suitability areas will expand northward, potentially exposing new temperate agricultural regions to climatic hospitality. It is crucial to emphasize that these suitability maps represent fundamental climatic constraints rather than realized invasion risk, as successful establishment is ultimately governed by host plant, land-use patterns, irrigation, and human-mediated transport. Furthermore, projections of future range shifts in this study are dependent on a single climate model (BCC-CSM2-MR) and should be viewed as potential trajectories rather than deterministic geographical forecasts. Maintaining strict prevention strategies and developing region-specific monitoring and Integrated Pest Management (IPM) measures are essential for mitigating the potential establishment and impact of *A. transitella* in China.

## Data Availability

The cleaned occurrence CSV (AT_cleaned.csv), analysis code (Step1.r, Step2.r, Step3.r), accessible area (M) shapefile (M.shp), Biomod2 ODMAP table, and final raster outputs are publicly available at GitHub: https://github.com/Li-PhDBio/Amyelois-transitella.R (accessed on 25 February 2026). The repository also documents the data processing workflow and model settings. Additional files and clarifications are available from the corresponding authors upon reasonable request.

## Abbreviations

The following abbreviations are used in this manuscript:

SDMs	Species Distribution Models
CMIP6	Coupled Model Intercomparison Project Phase 6
BCC-CSM2-MR	Beijing Climate Center Climate System Model version 2—Medium Resolution
SSP	Shared Socioeconomic Pathway
MaxEnt	Maximum Entropy Model
GLM	Generalized Linear Model
GAM	Generalized Additive Model
MARS	Multivariate Adaptive Regression Splines
ANN	Artificial Neural Network
CTA	Classification Tree Analysis
FDA	Flexible Discriminant Analysis
RF	Random Forest
SRE	Surface Range Envelope
ROC	Receiver Operating Characteristic
AUC	Area Under the ROC Curve
TSS	True Skill Statistic

## Appendix B

**Table A1:** Bioclimatic variables.

Code	Variable
BIO1	Annual Mean Temperature
BIO2	Mean Diurnal Range
BIO3	Isothermality
BIO4	Temperature Seasonality
BIO5	Max Temperature of Warmest Month
BIO6	Min Temperature of Coldest Month
BIO7	Temperature Annual Range
BIO8	Mean Temperature of Wettest Quarter
BIO9	Mean Temperature of Driest Quarter
BIO10	Mean Temperature of Warmest Quarter
BIO11	Mean Temperature of Coldest Quarter
BIO12	Annual Precipitation
BIO13	Precipitation of Wettest Month
BIO14	Precipitation of Driest Month
BIO15	Precipitation Seasonality
BIO16	Precipitation of Wettest Quarter
BIO17	Precipitation of Driest Quarter
BIO18	Precipitation of Warmest Quarter
BIO19	Precipitation of Coldest Quarter
elev	Elevation

**Table A2:** Contribution of variables after collinearity screening.

Variables	VIF
bio19	1.64689812726243
bio4	1.44161356097229
bio15	1.30637201995001
bio18	1.1465643375476

**Table A3:** Overview of modeling algorithms used in Biomod2 and their corresponding R packages.

Model	Overview (Principle/Application)	Dependent R Package in Biomod2
ANN	Artificial Neural Network. Mimics the biological neural system through nonlinear computation to learn complex relationships among variables. Suitable for modeling highly nonlinear species responses.	nnet v7.3-20
CTA	Classification Tree Analysis. Builds decision paths through recursive partitioning, suitable for discrete classification in variable space.	rpart v4.1.24
FDA	Flexible Discriminant Analysis. Combines nonparametric regression with linear discriminant analysis to handle nonlinear classification problems.	fda v6.3.0
GAM	Generalized Additive Model. Uses smooth functions to fit nonlinear relationships among variables and flexibly model both the response distribution and functional form.	gam v1.22-7, mgcv v1.9-4
GBM	Gradient Boosting Machine. Sequentially builds weak learners to fit residuals and combines them into a strong ensemble model effective for capturing complex interactions.	gbm v2.2.3
MARS	Multivariate Adaptive Regression Splines. Uses piecewise linear functions to model nonlinear relationships and capture interaction effects.	earth v5.3.5
MAXNET	Maximum Entropy Model. Estimates species suitability distribution by maximizing entropy, commonly used for presence-only data modeling	maxnet v0.1.4
RF	Random Forest. Constructs multiple decision trees and aggregates results through averaging or voting to improve prediction accuracy and robustness.	randomForest v4.7-1.2
SRE	Surface Range Envelope. Builds a simple climatic suitability envelope based on the upper and lower limits of environmental variables.	biomod2 v4.3-4-5
XGBOOST	Extreme Gradient Boosting. An enhanced gradient boosting model with optimization features such as regularization and parallel computation.	xgboost v3.2.1.1

## References

[1] Hulme P.E. (2022). Importance of Greater Interdisciplinarity and Geographic Scope When Tackling the Driving Forces behind Biological Invasions. Conserv. Biol..

[2] Panzavolta T., Bracalini M., Benigno A., Moricca S. (2021). Alien Invasive Pathogens and Pests Harming Trees, Forests, and Plantations: Pathways, Global Consequences and Management. Forests.

[3] Bragard C., Dehnen-Schmutz K., Di Serio F., Gonthier P., Jacques M.-A., Jaques Miret J.A., Justesen A.F., Magnusson C.S., Milonas P., EFSA Panel on Plant Health (PLH) (2021). Pest Categorisation of *Amyelois transitella*. EFSA J..

[4] Bragard C., Baptista P., Chatzivassiliou E., Di Serio F., Gonthier P., Jaques Miret J.A., Justesen A.F., MacLeod A., Magnusson C.S., EFSA Panel on Plant Health (PLH) (2022). Pest Risk Assessment of *Amyelois transitella* for the European Union. EFSA J..

[5] Mamo F.T., Abate B.A., Zheng Y., Nie C., He M., Liu Y. (2021). Distribution of Aspergillus Fungi and Recent Aflatoxin Reports, Health Risks, and Advances in Developments of Biological Mitigation Strategies in China. Toxins.

[6] Gordon P.E., Goodrich B.K., Wilson H. (2023). Adoption of *Amyelois transitella* (Navel Orangeworm) Monitoring and Management Practices across California Tree Nut Crops. J. Integr. Pest Manag..

[7] Siegel J.P. (2023). Nut Factors Associated with Navel Orangeworm, *Amyelois transitella* (Lepidoptera: Pyralidae) Damage to Pistachio (*Pistacia vera*) in California (2007–2017) and Implication for Control. J. Econ. Entomol..

[8] Siegel J.P. (2024). The Flight Pattern of Navel Orangeworm (*Amyelois transitella* Walker) 2008–2023 in California Pistachio. Insects.

[9] Baazeem A., Rodriguez A., Medina A., Magan N. (2021). Impacts of Climate Change Interacting Abiotic Factors on Growth, aflD and aflR Gene Expression and Aflatoxin B1 Production by Aspergillus Flavus Strains In Vitro and on Pistachio Nuts. Toxins.

[10] Zhang W., Dou J., Wu Z., Li Q., Wang S., Xu H., Wu W., Sun C. (2022). Application of Non-Aflatoxigenic Aspergillus Flavus for the Biological Control of Aflatoxin Contamination in China. Toxins.

[11] Suffert M., Wilstermann A., Petter F., Schrader G., Grousset F. (2018). Identification of New Pests Likely to Be Introduced into Europe with the Fruit Trade. EPPO Bull..

[12] Zhang H., Makowski D. (2025). Assessment of the Climatic Suitability for Invasive Agricultural Insect Pests in a Warming Europe Based on Köppen-Geiger Classification. Entomol. Gen..

[13] Ullah F., Zhang Y., Gul H., Hafeez M., Desneux N., Qin Y., Li Z. (2023). Estimation of the Potential Geographical Distribution of Invasive Peach Fruit Fly under Climate Change by Integrated Ecological Niche Models. CABI Agric. Biosci..

[14] Higbee B.S., Burks C.S. (2021). Individual and Additive Effects of Insecticide and Mating Disruption in Integrated Management of Navel Orangeworm in Almonds. Insects.

[15] Miliordos D.E., Baliota G.V., Athanassiou C.G., Natskoulis P.I. (2025). Review on the Occurrence of Mycotoxigenic Fungi in Dried Fruits and the Role of Stored-Product Insects. Toxins.

[16] Liu X., Huang W., Liu Y., Zhan A. (2024). Perspectives of Invasive Alien Species Management in China. Ecol. Appl..

[17] Qin Y., Zhang Y., Clarke A.R., Zhao Z., Li Z. (2021). Including Host Availability and Climate Change Impacts on the Global Risk Area of *Carpomya pardalina* (Diptera: Tephritidae). Front. Ecol. Evol..

[18] Azrag A.G.A., Mohamed S.A., Ndlela S., Ekesi S. (2023). Invasion Risk by Fruit Trees Mealybug *Rastrococcus invadens* (Williams) (Homoptera: Pseudococcidae) under Climate Warming. Front. Ecol. Evol..

[19] Elith J., Leathwick J.R. (2009). Species Distribution Models: Ecological Explanation and Prediction across Space and Time. Annu. Rev. Ecol. Evol. Syst..

[20] Barve N., Barve V., Jiménez-Valverde A., Lira-Noriega A., Maher S.P., Peterson A.T., Soberón J., Villalobos F. (2011). The Crucial Role of the Accessible Area in Ecological Niche Modeling and Species Distribution Modeling. Ecol. Modell..

[21] Soberon J., Peterson A.T. (2005). Interpretation of Models of Fundamental Ecological Niches and Species’ Distributional Areas. Biodivers. Inform..

[22] GBIF Occurrence Download.

[23] Baker D.J., Maclean I.M.D., Goodall M., Gaston K.J. (2022). Correlations between Spatial Sampling Biases and Environmental Niches Affect Species Distribution Models. Global Ecol. Biogeogr..

[24] Pires-Oliveira J.C., Bampi H., Lima-Ribeiro M.S., Eisenlohr P.V. (2023). Sampling Bias Worsen the Predictive Ability of Niche Models. Rev. Gest. Soc. Ambient..

[25] Pili A., Leroy B., Zurell D. (2025). Correcting Environmental Sampling Bias Improves Transferability of Species Distribution Models. Ecography.

[26] Braunisch V., Coppes J., Arlettaz R., Suchant R., Schmid H., Bollmann K. (2013). Selecting from Correlated Climate Variables: A Major Source of Uncertainty for Predicting Species Distributions under Climate Change. Ecography.

[27] Phillips S.J., Dudík M., Elith J., Graham C.H., Lehmann A., Leathwick J., Ferrier S. (2009). Sample Selection Bias and Presence-Only Distribution Models: Implications for Background and Pseudo-Absence Data. Ecol. Appl..

[28] Naimi B., Hamm N.A.S., Groen T.A., Skidmore A.K., Toxopeus A.G. (2014). Where Is Positional Uncertainty a Problem for Species Distribution Modelling?. Ecography.

[29] Lannuzel G., Balmot J., Dubos N., Thibault M., Fogliani B. (2021). High-Resolution Topographic Variables Accurately Predict the Distribution of Rare Plant Species for Conservation Area Selection in a Narrow-Endemism Hotspot in New Caledonia. Biodivers. Conserv..

[30] Zhou T., Chen Z., Zou L., Chen X., Yu Y., Wang B., Bao Q., Bao Y., Cao J., He B. (2020). Development of Climate and Earth System Models in China: Past Achievements and New CMIP6 Results. J. Meteorolog. Res..

[31] Li Y., Wang C., Su F. (2021). Evaluation of Climate in CMIP6 Models over Two Third Pole Subregions with Contrasting Circulation Systems. J. Clim..

[32] Ali Z., Iqbal M., Khan I.U., Masood M.U., Umer M., Lodhi M.U.K., Tariq M.A.U.R. (2023). Hydrological Response under CMIP6 Climate Projection in Astore River Basin, Pakistan. J. Mt. Sci..

[33] Fan X., Duan Q., Shen C., Wu Y., Xing C. (2020). Global Surface Air Temperatures in CMIP6: Historical Performance and Future Changes. Environ. Res. Lett..

[34] Ragab S.H., Tyshenko M.G., Halmy M.W.A. (2025). Impact of Climate Change on the Habitat Range of Monarch Butterfly (*Danaus plexippus*). Sci. Rep..

[35] Thuiller W., Lafourcade B., Engler R., Araújo M.B. (2009). BIOMOD—A Platform for Ensemble Forecasting of Species Distributions. Ecography.

[36] Thuiller W. (2003). BIOMOD—Optimizing Predictions of Species Distributions and Projecting Potential Future Shifts under Global Change. Glob. Change Biol..

[37] Petrosyan V., Dinets V., Osipov F., Dergunova N., Khlyap L. (2023). Range Dynamics of Striped Field Mouse (*Apodemus agrarius*) in Northern Eurasia under Global Climate Change Based on Ensemble Species Distribution Models. Biology.

[38] Wang T., Zhang T., An W., Wang Z., Li C. (2024). Predicting the Potential Geographic Distribution of Invasive Freshwater Apple Snail *Pomacea canaliculate* (Lamarck, 1819) under Climate Change Based on Biomod2. Agronomy.

[39] Wenger S.J., Olden J.D. (2012). Assessing Transferability of Ecological Models: An Underappreciated Aspect of Statistical Validation. Methods Ecol. Evol..

[40] Koç D.E., Atalay Dutucu A. (2024). Analyzing the Distribution Patterns of Endemic *Quercus vulcanica* (Boiss. et Heldr. Ex) Kotschy in Türkiye under Climate Change Using Ensemble Modeling. Forests.

[41] Adhikari P., Lee Y.H., Adhikari P., Hong S.H., Park Y.-S. (2022). Climate Change-Induced Invasion Risk of Ecosystem Disturbing Alien Plant Species: An Evaluation Using Species Distribution Modeling. Front. Ecol. Evol..

[42] Guo L., Gao Y., He P., He Y., Meng F. (2023). Modeling for Predicting the Potential Geographical Distribution of Three Ephedra Herbs in China. Plants.

[43] Su L., Hao Y., Xia J. (2025). Ensemble Model Projection of Climate-Driven Habitat Redistribution for the Endangered Brachymystax Tsinlingensis. J. Environ. Manag..

[44] Chen J., Yang S.T., Li H.W., Zhang B., Lv J.R. (2013). Research on Geographical Environment Unit Division Based on the Method of Natural Breaks (Jenks). Int. Arch. Photogramm. Remote Sens. Spat. Inf. Sci..

[45] Parrott L. (2017). The Modelling Spiral for Solving ‘Wicked’ Environmental Problems: Guidance for Stakeholder Involvement and Collaborative Model Development. Methods Ecol. Evol..

[46] Dormann C.F., Bobrowski M., Dehling D.M., Harris D.J., Hartig F., Lischke H., Moretti M.D., Pagel J., Pinkert S., Schleuning M. (2018). Biotic Interactions in Species Distribution Modelling: 10 Questions to Guide Interpretation and Avoid False Conclusions. Glob. Ecol. Biogeogr..

[47] Aiello-Lammens M.E., Boria R.A., Radosavljevic A., Vilela B., Anderson R.P. (2015). spThin: An R Package for Spatial Thinning of Species Occurrence Records for Use in Ecological Niche Models. Ecography.

[48] Otto C.R.V., Bailey L.L., Roloff G.J. (2013). Improving Species Occupancy Estimation When Sampling Violates the Closure Assumption. Ecography.

[49] Aviron S., Kindlmann P., Burel F. (2007). Conservation of Butterfly Populations in Dynamic Landscapes: The Role of Farming Practices and Landscape Mosaic. Ecol. Modell..

[50] Baldwin D.S., Fraser M. (2009). Rehabilitation Options for Inland Waterways Impacted by Sulfidic Sediments—A Synthesis. J. Environ. Manag..

[51] Elith J., Leathwick J.R., Hastie T. (2008). A Working Guide to Boosted Regression Trees. J. Anim. Ecol..

[52] Cutler D.R., Edwards T.C., Beard K.H., Cutler A., Hess K.T., Gibson J., Lawler J.J. (2007). Random Forests for Classification in Ecology. Ecology.

[53] Wood S.N. (2017). Generalized Additive Models: An Introduction with R.

[54] Davies S.C., Thompson P.L., Gomez C., Nephin J., Knudby A., Park A.E., Friesen S.K., Pollock L.J., Rubidge E.M., Anderson S.C. (2023). Addressing Uncertainty When Projecting Marine Species’ Distributions under Climate Change. Ecography.

[55] Eyring V., Bony S., Meehl G.A., Senior C.A., Stevens B., Stouffer R.J., Taylor K.E. (2016). Overview of the Coupled Model Intercomparison Project Phase 6 (CMIP6) Experimental Design and Organization. Geosci. Model Dev..

[56] Burks C.S., Higbee B.S., Brandl D.G., Mackey B.E. (2008). Sampling and Pheromone Trapping for Comparison of Abundance of *Amyelois transitella* in Almonds and Pistachios. Entomol. Exp. Appl..

[57] Sun Y., Deng Y., Yao S., Sun Y., Degen A.A., Dong L., Luo J., Xie S., Hou Q., Tang D. (2025). Distribution Range and Richness of Plant Species Are Predicted to Increase by 2100 Due to a Warmer and Wetter Climate in Northern China. Glob. Change Biol..

[58] Rossi J.-P., Mouttet R., Rousse P., Streito J.-C. (2024). Modelling the Potential Range of Agrilus Planipennis in Europe According to Current and Future Climate Conditions. Trees For. People.

[59] Guillén L., Pascacio-Villafán C., Osorio-Paz I., Ortega-Casas R., Enciso-Ortíz E., Altúzar-Molina A., Velázquez O., Aluja M. (2022). Coping with Global Warming: Adult Thermal Thresholds in Four Pestiferous Anastrepha Species Determined under Experimental Laboratory Conditions and Development/Survival Times of Immatures and Adults under Natural Field Conditions. Front. Physiol..

[60] Jałoszyński P. (2015). The Cephenniini of China. VI. New Species and New Records of Cephennodes Reitter from Hainan, Guangxi and Guangdong (Coleoptera: Staphylinidae: Scydmaeninae). Zootaxa.

[61] Liu M., Wang X., Zhang Y., Xu L., Liu Y., Yu L., Ma F., Wang X., Gong Z., Zhang L. (2023). Chemical Composition of Walnuts from Three Regions in China. Oil Crop Sci..

[62] Musolin D.L., Kirichenko N.I., Karpun N.N., Aksenenko E.V., Golub V.B., Kerchev I.A., Mandelshtam M.Y., Vasaitis R., Volkovitsh M.G., Zhuravleva E.N. (2022). Invasive Insect Pests of Forests and Urban Trees in Russia: Origin, Pathways, Damage, and Management. Forests.

[63] Raffa K.F., Brockerhoff E.G., Grégoire J.-C., Hamelin R.C., Liebhold A.M., Santini A., Venette R.C., Wingfield M.J. (2023). Approaches to Forecasting Damage by Invasive Forest Insects and Pathogens: A Cross-Assessment. Bioscience.

[64] Timm A.E., Tembrock L.R., Zink F.A., Mollet K.A. (2025). A Real-Time PCR Assay for Detecting Codling Moth Cydia Pomonella on Material Intercepted at U.S. Ports of Entry—A Valuable Tool for Specimen Identification. Int. J. Mol. Sci..

[65] Graham D., Anyamba A., Davison B., Martin S., Petoskey B., Rush T., Weston D., Yang X. (2024). Plant Disease Detection Technology Assessment.

[66] Deliveyne N., Young J.M., Austin J.J., Cassey P. (2023). Shining a LAMP on the Applications of Isothermal Amplification for Monitoring Environmental Biosecurity. NeoBiota.

[67] EFSA Panel on Plant Health (PLH) (2012). Guidance on Methodology for Evaluation of the Effectiveness of Options for Reducing the Risk of Introduction and Spread of Organisms Harmful to Plant Health in the EU Territory. EFSA J..

[68] Broadhead G.T., Higbee B.S., Beck J.J. (2025). Evaluating the Use of In-Season Measures of Pest Abundance to Predict End-of-Season Damage: A Study in Commercial Almond (*Prunus dulcis*). Pest Manag. Sci..

